# Interaction of Proteins with Biomembranes

**DOI:** 10.3390/membranes12020181

**Published:** 2022-02-02

**Authors:** Yosuke Senju, Shiro Suetsugu

**Affiliations:** 1 Research Institute for Interdisciplinary Science (RIIS), Okayama University, Okayama 700-8530, Japan; 2 Division of Biological Science, Graduate School of Science and Technology, Nara Institute of Science and Technology, Nara 630-0192, Japan; 3 Data Science Center, Nara Institute of Science and Technology, Nara 630-0192, Japan; 4 Center for Digital Green-innovation, Nara Institute of Science and Technology, Nara 630-0192, Japan

Many proteins interact with cell and subcellular membranes. The plasma and intracellular membranes are characterized by their different lipid compositions that enable membrane-binding proteins to localize to distinct subcellular compartments. These lipid–protein interactions also regulate protein conformation and protein–protein interactions, which precisely regulate the activation of molecular complexes at the respective membranes. Furthermore, membrane-bound proteins can control lipid lateral diffusion, membrane tension/fluidity, and lipid phase separation. These membrane properties can induce the intracellular signaling that plays crucial roles in various cellular processes such as cell migration, morphogenesis, membrane trafficking, and signal transduction. However, due to the complexity of the abundant protein–protein interactions within a cell, the exact molecular mechanisms underlying protein interactions with lipids contain a lot of unclarity.

This *Membranes* Special Issue, entitled “Interaction of Proteins with Biomembrane”, discusses the recent progress in lipid–protein interactions from various perspectives, including cell biology, biochemistry, and biophysics. These studies will elucidate the mechanisms by which membranes regulate protein localizations/functions, and thus provide new insights into the fundamental principles of lipid–protein interactions. A summary of the research articles is presented here.

Motegi et al. [1] studied the formation of phosphatidylinositol (PI)-induced microdomains on supported lipid bilayers using atomic force microscopy (AFM) and single-particle tracking. The authors found that the PI-induced microdomains had less fluidity than the surrounding regions where lipids freely diffused, and thus functioning as diffusion barriers. These PI-induced microdomains acted as a scaffold to promote the initial clustering of FBP17, one of the membrane-remodeling Bin/Amphiphysin/Rvs (BAR) domain proteins. The surrounding fluid region promoted FBP17 assembly through lipid lateral diffusion. This study suggests the possible role of lipid microdomains in the self-assembly of membrane-binding proteins according to their lipid composition and physical properties.

To et al. [2] studied the non-structural (NS) protein, NS4A, a membrane protein critical for virulence, and thus flavivirus membrane morphogenesis. The authors found that a peptide containing an N-terminal cytoplasmic tail and one-third of the first transmembrane domain of Zika virus (ZIKV) NS4A formed homotrimers. The authors propose that the disruption of this oligomerization is essential for in vitro screening assays for the antiviral discovery.

By using a single quantum dot tracking approach, Kovtun et al. [3] studied the lateral diffusion, nanodomain formation, and their implications in signal transduction of the D2 subtype dopamine receptor (D2DR), a class A G-protein-coupled receptor (GPCR), which has naturally occurring genetic variants in schizophrenia. The authors found a significant decrease in the diffusion dynamics of the Val96Ala D2L schizophrenia variant. By measuring the relative frequency of D2L–D2L interactions, the authors found a significant fraction of D2L receptors and their variants transiently colocalized. The authors also compared nanoclusters of D2DR to those of phosphatidylinositol 4,5-bisphosphate in the plasma membrane.

Aalst et al. [4] studied the cholesterol binding of the CC motif chemokine receptor 3 (CCR3), a class A GPCR, which is mainly responsible for the cellular trafficking in eosinophils. CCR3 plays vital roles in inflammatory conditions such as asthma, arthritis and the cancer metastasis. The authors analyzed lipid–protein contacts to identify the potential cholesterol-binding sites in the transmembrane region of CCR3 by using in silico coarse-grained molecular dynamics (MD) using PyLipID. Several cholesterol-binding sites of CCR3 contain a cholesterol recognition/interaction amino acid consensus (CRAC) motif and its inverted CARC motif. Generally, the CARC motif in the transmembrane region is located in the outer membrane leaflet, and its mirror motif, CRAC, is in the inner membrane leaflet. Based on the sequence alignment, these cholesterol-binding sites are conserved not only in CCR3 but also in other CC and CXC motif chemokine receptors. Furthermore, the functional residues in and near these sites were implicated in receptor dimerization, ligand binding, and signal transduction. The authors propose that their findings provide insights into the mechanisms underlying cholesterol regulation of the class A GPCR subfamily.

Taken together, the papers in this Special Issue will update our current knowledge of the interaction between proteins and biomembranes. The technical approaches presented here, such as AFM, single-particle tracking, and supported lipid bilayers, will help us understand the spatiotemporal dynamics of protein/lipid lateral diffusion, compartmentalization of the cell membrane, and microdomain formations and their physical properties. These studies will provide new insights into the fundamental principles underlying physiological functions of membrane proteins such as GPCRs and membrane-remodeling proteins in cells and tissues.
